# Exploring the relationship between novel serum inflammatory markers, non-traditional lipid parameters, and in-stent restenosis after percutaneous coronary intervention: a single-center retrospective study

**DOI:** 10.3389/fcvm.2026.1803923

**Published:** 2026-07-09

**Authors:** Mingliang Du, Miao Jiang, Hui Hui

**Affiliations:** Department of Coronary Heart Disease, Central Hospital of Dalian University of Technology, (Dalian Municipal Central Hospital), Dalian, China

**Keywords:** coronary heart disease, in-stent restenosis, non-traditional lipid parameters, novel serum inflammatory markers, percutaneous coronary intervention

## Abstract

**Background:**

Despite advances in drug-eluting stents (DES), in-stent restenosis (ISR) remains a significant cause of percutaneous coronary intervention (PCI) failure. Inflammation and lipid metabolism are implicated in ISR pathogenesis. This study aimed to investigate the correlation and predictive value of novel systemic inflammatory indices and non-traditional lipid parameters for ISR after DES implantation.

**Methods:**

This single-center, retrospective study enrolled 564 patients who underwent initial DES implantation and received follow-up coronary angiography at least six months later. Patients were divided into ISR (*n* = 112) and non-ISR (*n* = 452) groups. Clinical data and laboratory parameters were collected. Various inflammatory indices (SII, SIRI, NLR, PLR, MLR, NPR, PIV) and non-traditional lipid parameters (AIP, AC, CRI-I, CRI-II, LCI, RC) were calculated. Univariate and multivariate logistic regression analyses were performed to identify factors independently associated with ISR. Receiver operating characteristic (ROC) curve analysis was used to evaluate the predictive performance of significant indicators.

**Results:**

Compared to the non-ISR group, the ISR group had significantly higher levels of SII, SIRI, NLR, PLR, MLR, PIV, CRI-II, and LCI (all *P* < 0.05). Adjusted logistic regression identified CRI-II (OR =  1.277, 95% CI: 1.066–1.529, *p* = 0.008) and LCI (OR = 1.010, 95% CI: 1.002–1.018, *p* = 0.020) as independent risk factors.ROC analysis showed that CRI-II had an AUC of 0.586 (*P* = 0.005) with a sensitivity of 75.0% and a specificity of 41.2%, while LCI demonstrated an AUC of 0.571 (*P* = 0.020) with a sensitivity of 58.0% and a specificity of 55.8%.None of the novel serum inflammatory markers were independently associated with ISR in the multivariate logistic regression analysis.

**Conclusion:**

CRI-II and LCI, are significantly associated with ISR after PCI but show limited discriminatory ability. All AUC values were below 0.7, indicating these findings are exploratory signals only. These easily accessible biomarkers exhibit statistical associations but are not ready for clinical recommendation for ISR risk stratification.

## Introduction

Currently, drug-eluting stents (DES) constitute the primary therapeutic approach for managing coronary heart disease (CHD), significantly enhancing patient outcomes. Due to advancements in stent technology and improved antiplatelet therapies, the rate of in-stent restenosis (ISR) within the first year post-percutaneous coronary intervention (PCI) has declined markedly, from approximately 20%–30% with bare-metal stents to roughly 5%–10% today. The predominant mechanism underlying ISR involves vascular smooth muscle cell (VSMC) proliferation and the subsequent formation of neointimal tissue (1). Recurrent cardiovascular events, such as sudden cardiac death and myocardial infarction, are often linked to ISR. Recent studies have shown that ISR cases associated with DES clinically manifest as unstable angina in 27%–50% of cases, and acute myocardial infarction in approximately 5%–11% of affected patients. Consequently, symptoms frequently recur, necessitating repeated revascularization and highlighting the unsuccessful outcome of PCI (2). Therefore, the early and accurate identification of practical biomarkers for ISR has significant clinical importance for CHD patients.

Inflammation is closely associated with cardiovascular diseases. After stent implantation, inflammatory responses significantly influence ISR progression during both acute and chronic phases. Mechanical injury to the vascular wall from stent implantation triggers a local inflammatory reaction, attracting inflammatory cells to the injury site. These cells release inflammatory mediators, which stimulate VSMC proliferation and migration, ultimately causing neointimal hyperplasia (3). Previous studies have highlighted that routinely accessible and cost-effective hematological parameters, such as white blood cell (WBC) counts and specific subtypes like neutrophils and monocytes, exhibit correlations with cardiovascular event risk, including myocardial infarction (4). Additionally, several novel inflammatory biomarkers have recently been introduced, such as systemic immune-inflammation index (SII), systemic inflammatory response index (SIRI), neutrophil-lymphocyte ratio (NLR), platelet-to-lymphocyte ratio (PLR), monocyte-to-lymphocyte ratio (MLR), neutrophil-platelet ratio (NPR), and pan-immune inflammation value (PIV). These indicators integrate multiple blood cell subtype ratios, thereby reflecting the comprehensive inflammatory and immune state of the patient.

Among traditional lipid parameters, Low-density lipoprotein cholesterol(LDL-C) is well-established as a major factor in cardiovascular disease. All global guidelines for dyslipidemia management identify LDL-C as the primary lipid-lowering therapeutic target for both primary and secondary cardiovascular prevention. However, clinical pharmacological interventions focusing solely on this marker indicate that even when LDL-C levels reach target values, a substantial residual risk of cardiovascular events remains. Thus, lowering LDL-C alone does not fully address cardiovascular prevention needs (5). Consequently, non-traditional lipid parameters have emerged, such as the atherogenic index of plasma (AIP), atherogenic coefficient (AC), Castelli's risk index-I (CRI-I), Castelli's risk index-II (CRI-II), lipoprotein combined index (LCI), and remnant cholesterol (RC). However, few studies have explored the relationship between these parameters and ISR.

In summary, investigating novel serum inflammatory markers and non-traditional lipid parameters (which are cost-effective and convenient) in relation to ISR is essential for effectively assessing vascular status and improving CHD prognosis.

Previous studies have separately explored the roles of inflammatory indices such as NLR, PLR, and SII in ISR, but few have simultaneously evaluated a large panel of novel inflammatory markers including SIRI and PIV together with a comprehensive panel of non-traditional lipid parameters in a real-world Chinese cohort.

Therefore, the present study is designed to fill this gap by comprehensively comparing the predictive values of both inflammatory and lipid indices, which may provide a more complete understanding of risk factors for ISR.

## Materials and methods

### Study population

This retrospective observational study evaluated data from 564 patients who received their initial DES implantation at Dalian University of Technology (Dalian Municipal Central Hospital) from January 2020 to January 2025.All included patients underwent at least one follow-up coronary angiography (CAG) conducted more than six months post-procedure. Of these patients, 439 were males, and 125 were females. Blood samples were collected within 24 h before PCI for laboratory measurements.

Inclusion criteria: (1) patients diagnosed with CHD by CAG at Dalian University of Technology (Dalian Municipal Central Hospital) who underwent their first DES implantation; (2) patients who either planned routine follow-up CAG or returned due to symptoms such as chest tightness, chest pain, or other discomforts, with re-examination occurring more than six months after the initial procedure; (3) patients with complete surgical records; (4) patients who adhered to regular postoperative use of antiplatelet drugs, lipid-lowering medications, and other treatments.

Exclusion criteria: (1) patients with hematological disorders, autoimmune diseases, or malignant tumors; (2) patients with active infections prior to surgery; (3) patients with severe liver or kidney failure; (4) patients with severe heart failure, valvular heart disease, or history of cardiac surgery.

This retrospective observational research, performed at a single institution, strictly complied with the ethical standards stipulated in the Declaration of Helsinki. Ethical clearance was obtained from the Ethics Committee of Dalian University of Technology (Dalian Municipal Central Hospital),under the reference number YN2026-066-01. Patient clinical baseline data were obtained retrospectively from medical records.

### Surgical information

All patients underwent coronary angiography and DES implantation in the cardiac catheterization laboratory of Dalian University of Technology (Dalian Municipal Central Hospital). Branch stenoses of major coronary vessels were assigned to the corresponding parent vessels. All implanted stents were second-generation DESs. Procedural data were collected, including stent implantation sites, the total number of implanted stents, and detailed operative records. Based on angiographic evaluations conducted during follow-up, 564 patients who met the specified inclusion and exclusion criteria were divided into ISR and non-ISR groups. ISR was defined explicitly as a new lesion appearing either within the stented segment or extending up to 5 mm beyond the proximal or distal stent margins, leading to vessel lumen narrowing exceeding 50% (6).

### Calculation of derived indices

All inflammatory and lipid indices were calculated using the following formulas (Equations 1–13):

$$SII\;=\frac{\;platelet\;\mathrm{count}\;\times \;neutrophil\;\mathrm{count}}{\;lymphocyte\;\mathrm{count}}$$ (1)

$$SIRI\;=\frac{monocyte\;\mathrm{count}\;\times \;neutrophil\;\mathrm{count}}{\;lymphocyte\;\mathrm{count}}$$ (2)

$$NLR\;=\frac{\;neutrophil\;\mathrm{count}\;}{\;lymphocyte\;\mathrm{count}}$$ (3)

$$PLR\;=\frac{\;platelet\;\mathrm{count}}{lymphocyte\;\mathrm{count}}$$ (4)

$$MLR=\frac{\;monocyte\;\mathrm{count}\;}{lymphocyte\;\mathrm{count}}$$ (5)

$$NPR=\frac{neutrophil\;\mathrm{count}}{\;platelet\;\mathrm{count}\;}$$ (6)

$$PIV=\frac{neutrophil\;\mathrm{count}\;\times monocyte\;\mathrm{count}\;\times platelet\;\mathrm{count}}{lymphocyte\;\mathrm{count}}$$ (7)

$$AIP={\text{log}}_{10}\frac{TG\;}{\;HDL-C}$$ (8)

$$AC=\frac{non-HDL-C}{\;HDL-C}$$ (9)

$$CRI-I=\frac{TC}{HDL-C}$$ (10)

$$CRI-II=\frac{\;LDL-C}{HDL-C}$$ (11)

$$LCI=\frac{TC\;\times \;TG\;\times \;LDL-C}{HDL-C\;}$$ (12)

RC=TC−HDL−C−LDL−C (13)

### Statistical analysis

SPSS software (version 26.0) facilitated statistical analyses. Normally distributed continuous data were expressed as means ± standard deviation (SD), while non-normally distributed data were represented as median values with interquartile ranges [M (P25, P75)]. The t-test or Mann–Whitney U test was employed for between-group comparisons, depending on the normality of the data. Categorical variables were expressed as counts and percentages [n (%)], and comparisons between groups were conducted using the chi-square test. Logistic regression analysis was used to identify factors associated with ISR, and receiver operating characteristic (ROC) curve analysis was applied to evaluate the predictive value of independently associated factors. To address multicollinearity and prevent model overfitting, indicators with significant differences in baseline characteristics were designated as adjusting variables. Separate logistic regression analyses were then performed for each of the eight key biochemical indicators individually. *P* < 0.05 was considered statistically significant.

## Results

### Baseline characteristics

**Figure 1:**
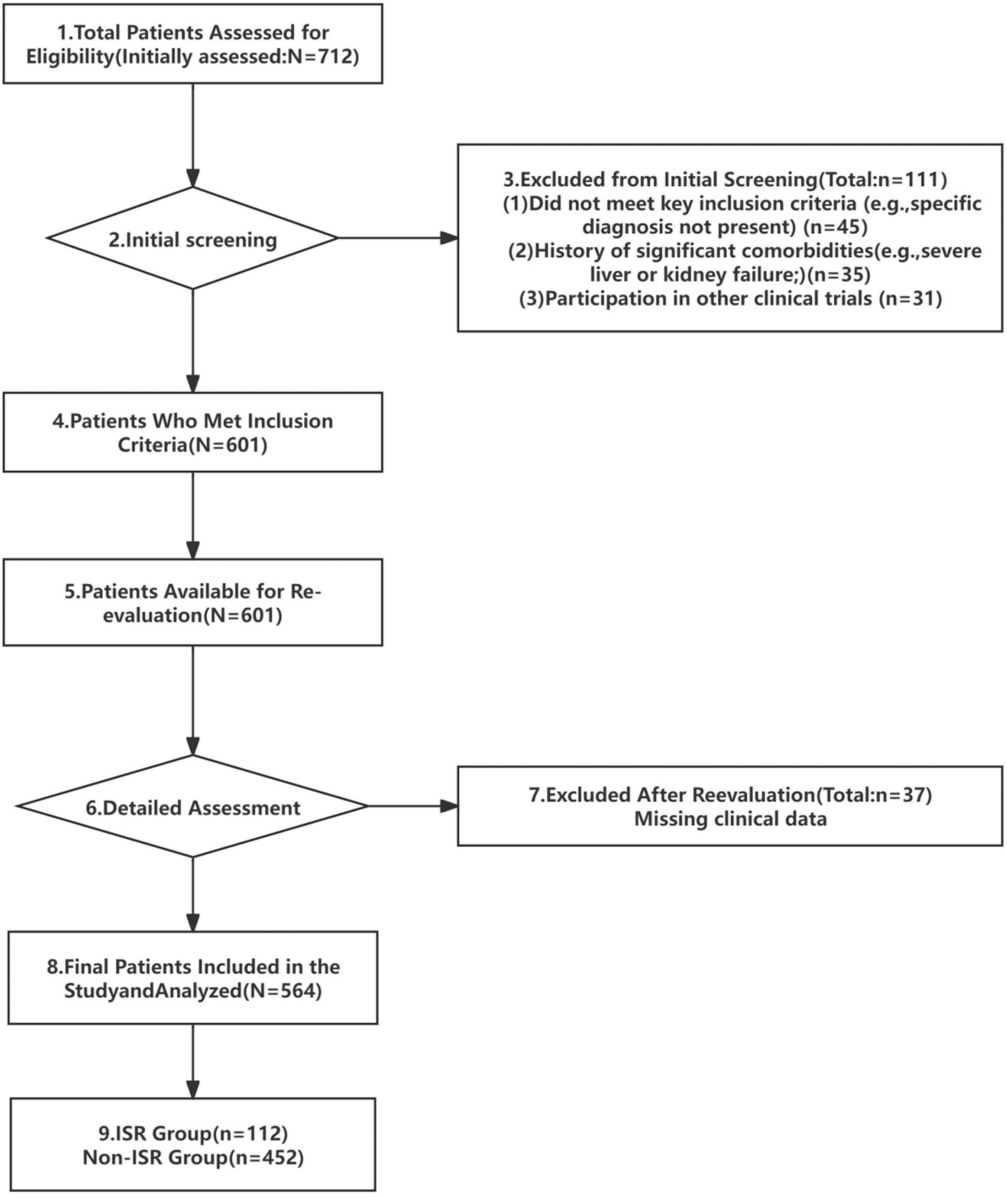
Patient enrollment flowchart.

In total, 564 patients were analyzed and allocated into ISR (*n* = 112) and non-ISR groups (*n* = 452) based on follow-up angiography outcomes. Comparative analyses of baseline parameters revealed significant differences in age, ALT, AST, creatinine, hemoglobin, WBC, red blood cell count (RBC), TC, LDL-C, fasting blood glucose (FBG), systolic blood pressure, diastolic blood pressure, and weight (*P* < 0.05). Patients in the ISR group exhibited higher age, AST levels, creatinine, WBC, TC, LDL-C, and FBG (*P* < 0.05). Conversely, the ISR group demonstrated significantly lower ALT, hemoglobin, RBC count, systolic and diastolic blood pressures, and body weight (*P* < 0.05) (Figure 1 and Table 1).

**Table 1:** Comparison of baseline characteristics between the ISR and Non-ISR groups.

Variable		Non-ISR (*n* = 452)	ISR (*n* = 112)	X^2^/Z	P
Sex	Male	347 (76.77)	92 (82.14)	1.502	0.220
	Female	105 (23.23)	20 (17.86)		
Smoking history	No	286 (63.27)	59 (52.68)	4.243	0.039
	Yes	166 (36.73)	53 (47.32)		
Alcohol consumption history	No	378 (83.63)	88 (78.57)	1.599	0.206
	Yes	74 (16.37)	24 (21.43)		
Hypertension	No	141 (31.19)	31 (27.68)	0.524	0.469
	Yes	311 (68.81)	81 (72.32)		
Diabetes mellitus	No	264 (58.41)	64 (57.14)	0.059	0.808
	Yes	188 (41.59)	48 (42.86)		
Stent implantation sites	LM	18 (3.98)	3 (2.68)	3.643	0.303
	LAD	217 (48.01)	45 (40.18)		
	LCX	81 (17.92)	27 (24.11)		
	RCA	136 (30.09)	37 (33.04)		
Number of diseased blood vessels				0.631	0.729
1 vessel		183 (40.49)	42 (37.5)		
2vessels		148 (32.74)	36 (32.14)		
3vessels		121 (26.77)	34 (30.36)		
Number of stents		2.74 ± 1.38	2.60 ± 1.44	0.953	0.341
stent diameter（mm）		3.09 ± 0.37	3.02 ± 0.42	1.642	0.103
Length of stent（mm）		22.91 ± 4.88	23.37 ± 5.78	−0.846	0.398
Gensini score		45.86 ± 21.54	49.23 ± 22.07	−1.476	0.141
Age		68 (61,73)	70.5 (66,76.75)	−3.218	0.001
Systolic blood pressure		135 (124,150)	131 (120,146)	−2.190	0.029
Diastolic blood pressure		80 (72,88)	74 (67,81.75)	−4.966	<0.001
Pulse		71 (67.25,80)	72 (68,75)	−0.055	0.956
Height		170 (164.75,175)	170 (166,175)	−0.793	0.428
Weight		75 (67,82.13)	72 (65,80)	−2.941	0.003
ALT		26 (18,37)	18 (14,28)	−4.037	<0.001
AST		23 (18,32)	26.5 (18,42.75)	−2.298	0.022
D-dimer		0.59 (0.46,0.77)	0.58 (0.33,0.83)	−0.455	0.649
Creatinine		75 (62.45,93.7)	85 (70.5,121)	−4.358	<0.001
Hemoglobin		132 (111,145)	124 (100,138)	−2.752	0.006
WBC		6.68 (5.37,8.27)	7.27 (6.1,9.72)	−2.837	0.005
RBC		4.4 (3.8,4.77)	4.12 (3.53,4.6)	−3.059	0.002
Platelet count		199.5 (162,246.5)	208.5 (165.75,262.75)	−1.936	0.053
TC		3.3 (2.71,4.08)	3.7 (2.93,4.31)	−2.676	0.007
LDL-C		1.77 (1.2,2.39)	2.13 (1.54,2.77)	−3.523	<0.001
TG		1.27 (0.95,1.83)	1.37 (1.03,1.86)	−0.810	0.418
HDL-C		0.86 (0.74,1.04)	0.92 (0.76,1.1)	−1.559	0.119
Fasting blood glucose		5.8 (5.08,7.24)	6.29 (5.26,8.61)	−2.956	0.003
Total protein		65.55 (62.05,69.6)	66 (63.35,71.9)	−1.842	0.065
Albumin		40.1(37.83,42.68)	39.95(37.93,42)	−1.109	0.267

ISR, in-stent restenosis; ALT, alanine aminotransferase; AST, aspartate aminotransferase; WBC, white blood cell; RBC, red blood cell; TC, total cholesterol; LDL-C, low-density lipoprotein cholesterol; TG, triglyceride; HDL-C, high-density lipoprotein cholesterol; FBG, fasting blood glucose; LM, left main coronary artery; LAD, left anterior descending artery; LCX, left circumflex artery; RCA, right coronary artery.

### Comparison of derived indices between the two groups

Since none of the derived indices showed normal distribution, analysis utilized the Mann–Whitney U test. Significant differences emerged between ISR and non-ISR groups regarding eight indices: SII, SIRI, NLR, PLR, MLR, PIV, CRI-II, and LCI (*P* < 0.05). All these indices were notably higher in the ISR group. However, no significant differences were observed for NPR, AIP, AC, CRI-I, and RC (*P* > 0.05) (Table 2).

**Table 2:** Comparison of various parameters between the two groups.

Variable	Non-ISR (*n* = 452)	ISR group (*n* = 112)	Z	P
SII	522.25 (356.86,924.64)	699.71 (481.48,1101.72)	−3.620	<0.001
SIRI	1.29 (0.73,2.33)	1.68 (1.05,3.58)	−3.353	0.001
NLR	2.75 (1.98,4.28)	3.21 (2.41,5.51)	−2.748	0.006
PLR	125.56 (94.28,179.58)	144.23 (104.86,185.93)	−2.137	0.033
MLR	0.3 (0.21,0.44)	0.35 (0.25,0.5)	−2.767	0.006
NPR	0.02 (0.02,0.03)	0.02 (0.02,0.03)	−0.818	0.413
PIV	243.27 (128.12,480.28)	389.54 (201.07,656.44)	−4.204	<0.001
AIP	0.16 (0,0.35)	0.17(−0.01,0.37)	−0.108	0.914
AC	2.8 (2.08,3.67)	3.01 (2.21,4)	−1.509	0.131
CRI-I	3.8 (3.08,4.67)	4.01 (3.21,5)	−1.509	0.131
CRI-II	2.03 (1.44,2.74)	2.38 (1.78,3.16)	−2.835	0.005
LCI	8.59 (4.07,18.56)	11.05 (5.41,24.46)	−2.325	0.020
RC	0.59 (0.42,0.79)	0.53 (0.35,0.77)	−1.396	0.163

SII, systemic immune-inflammation index; SIRI, systemic inflammatory response index; NLR, neutrophil-lymphocyte ratio; PLR, platelet-to-lymphocyte ratio; MLR, monocyte-to-lymphocyte ratio; NPR, neutrophil-platelet ratio; PIV, pan-immune inflammation value; AIP, atherogenic index of plasma; AC, atherogenic coefficient; CRI-I, Castelli's risk index I; CRI-II, Castelli's risk index II; LCI, lipoprotein combined index; RC, remnant cholesterol.

### Adjusted logistic regression analysis

Multivariate logistic regression analysis was performed to evaluate the independent predictive value of key indicators for ISR. The model construction adhered to the following rigorous criteria: First, clinical characteristics showing statistically significant differences (*P* < 0.05) in the univariate analysis (Table 1) were selected as candidate confounding variables. Second, to avoid structural multicollinearity that could distort regression coefficients, the constituent numerator and denominator parameters (e.g., TC, TG, LDL-C, and HDL-C) were strictly excluded from the multivariate models when analyzing their derivative ratio-based indicators (e.g., CRI-II and LCI). Finally, using the remaining significant (Table 1) variables as covariates for adjustment, each of the eight primary indicators from (Table 2) was entered into separate regression models one by one. It was found that only CRI-II and LCI were specifically correlated with ISR and the adjusted odds ratios and their corresponding 95% confidence intervals (95% CI) were calculated (Tables 3, 4).

**Table 3:** Logistic regression analysis of positive influencing factors.

Variable	B	Standard Error	Wald *χ*^2^	P	OR	95% CI
Smoking history	0.564	0.239	5.576	0.018	1.758	1.101∼2.808
Age	0.021	0.012	3.301	0.069	1.021	0.998∼1.045
Systolic blood pressure	0.006	0.007	0.704	0.401	1.006	0.993∼1.019
Diastolic blood pressure	−0.053	0.013	16.402	0.000	0.949	0.925∼0.973
Weight	−0.026	0.011	5.860	0.015	0.974	0.954∼0.995
ALT	0.000	0.003	0.002	0.969	1.000	0.995∼1.006
AST	0.003	0.002	3.422	0.064	1.003	1.000∼1.007
Creatinine	0.001	0.001	1.471	0.225	1.001	0.999∼1.003
Hemoglobin	0.000	0.005	0.004	0.952	1.000	0.990∼1.010
WBC	0.033	0.035	0.875	0.350	1.033	0.965∼1.106
RBC	−0.286	0.187	2.334	0.127	0.751	0.520∼1.084
Fasting blood glucose	0.112	0.039	8.326	0.004	1.118	1.036∼1.206
CRI-II	0.244	0.092	7.049	0.008	1.277	1.066∼1.529

ALT, alanine aminotransferase; AST, aspartate aminotransferase; WBC, white blood cell; RBC, red blood cell; CRI-II, Castelli's risk index II.

**Table 4:** Logistic regression analysis of positive influencing factors.

Variable	B	Standard Error	Wald χ^2^	P	OR	95% CI
Smoking history	0.582	0.239	5.933	0.015	1.789	1.120∼2.857
Age	0.021	0.012	3.316	0.069	1.021	0.998∼1.045
Systolic blood pressure	0.006	0.007	0.724	0.395	1.006	0.993∼1.019
Diastolic blood pressure	−0.054	0.013	16.803	0.000	0.948	0.924∼0.972
Weight	−0.026	0.011	5.712	0.017	0.974	0.954∼0.995
ALT	0.000	0.003	0.001	0.979	1.000	0.994∼1.005
AST	0.003	0.002	3.156	0.076	1.003	1.000∼1.007
Creatinine	0.001	0.001	1.562	0.211	1.001	0.999∼1.003
Hemoglobin	0.000	0.005	0.000	0.999	1.000	0.990∼1.010
WBC	0.031	0.035	0.789	0.374	1.031	0.964∼1.104
RBC	−0.298	0.186	2.557	0.110	0.742	0.515∼1.070
Fasting blood glucose	0.116	0.039	8.840	0.003	1.122	1.040∼1.211
LCI	0.010	0.004	5.386	0.020	1.010	1.002∼1.018

ALT, alanine aminotransferase; AST, aspartate aminotransferase; WBC, white blood cell; RBC, red blood cell; LCI, lipoprotein combined index.

### Predictive value of CRI-II and LCI for ISR

**Figure 2:**
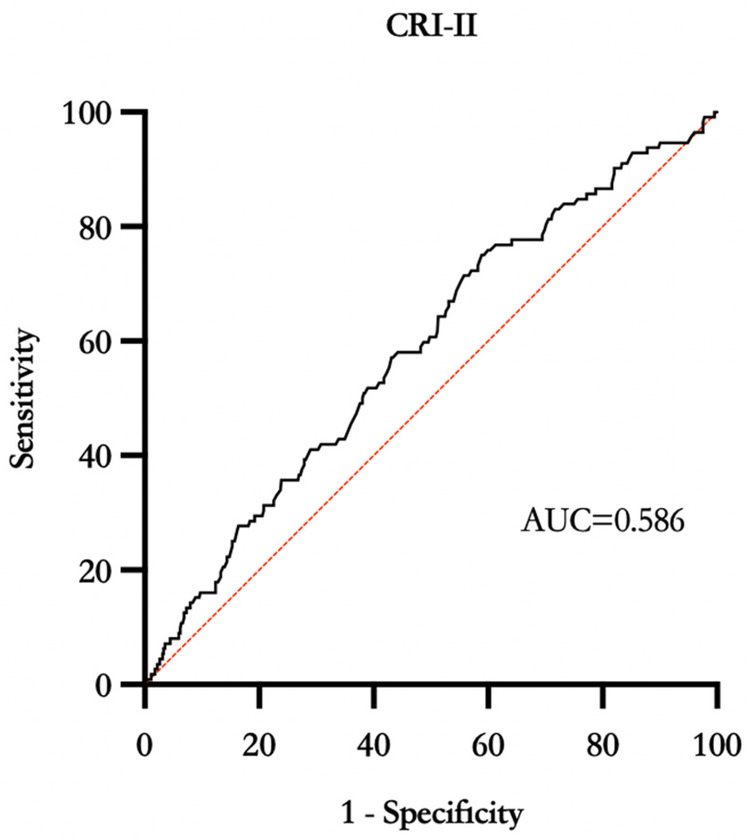
ROC curve of CRI-II for the diagnosis of ISR. ROC, receiver operating characteristic; CRI-II, Castelli’s risk index II.

**Figure 3:**
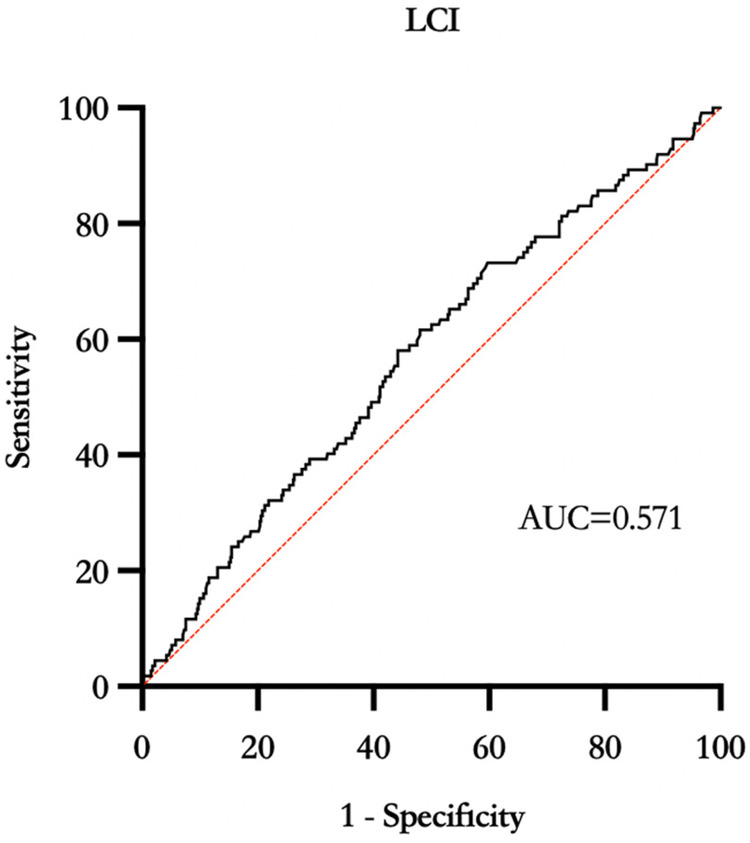
ROC curve of LCI for the diagnosis of ISR. ROC, receiver operating characteristic; LCI, lipoprotein combined index.

ROC curve analysis was employed to determine the predictive accuracy of CRI-II and LCI for ISR (Table 5 and Figures 2, 3).

**Table 5:** Diagnostic performance of each factor for ISR.

Variable	Youden Index	AUC	Sensitivity	Specificity	Optimal Cutoff Value	P
CRI-II	0.162	0.586	0.750	0.412	1.795	0.005
LCI	0.138	0.571	0.580	0.558	9.870	0.020

AUC, area under the curve; CRI-II, Castelli's risk index II; LCI, lipoprotein combined index.

## Discussion

Despite significant advances in contemporary stent technologies, identifying reliable biomarkers to predict ISR remains challenging for interventional cardiologists worldwide. The present study is the first to simultaneously evaluate a series of novel inflammatory indices and multiple non-traditional lipid parameters for ISR in a Chinese cohort undergoing PCI with DES. Such biomarkers enable effective risk stratification and timely interventions, ultimately improving clinical outcomes. This retrospective study investigated associations between novel serum inflammatory indices, non-traditional lipid parameters, and ISR occurrence after PCI. The primary findings indicated that CRI-II and LCI were significantly associated with ISR. Although neither of the two indices achieved an AUC above 0.7（CRI-II：0.586；LCI：0.571）, such moderate discrimination is common in exploratory biomarker studies, suggesting limited individual predictive capacity.

CRI-II, originally proposed by Castelli et al. (7), has been recognized as a robust predictor of coronary heart disease (CHD). Since then, numerous studies globally have explored the potential clinical utility of these indices. Notably, the identification of CRI-II as an independent predictor of ISR highlights the clinical superiority of composite lipid indices over single parameters.The mechanistic basis underlying this association likely involves the following interconnected pathways:(1) Endothelial barrier disruption: Balloon dilation and stent implantation during percutaneous coronary intervention (PCI) cause mechanical denudation and laceration of the vascular endothelium, compromising its barrier function. Consequently, LDL-C (serving as the numerator) readily penetrates the damaged endothelium into the subendothelial space, where it is retained by matrix components and undergoes oxidative modification (ox-LDL) (8).Concurrently, HDL-C (serving as the denominator) mediates reverse cholesterol transport, trafficking cholesterol from peripheral tissues—including within vascular plaques—back to the liver for clearance. An elevated CRI-II indicates that the local lipid influx and deposition rate far exceeds the HDL-mediated clearance and efflux rate, culminating in the uncontrolled accumulation of lipids around the stented segment (9).(2) Foam cell formation and inflammatory remodeling: The ox-LDL deposited locally at the stent site activates endothelial cells to release chemokines. This promotes the migration of monocytes into the subendothelial space, where they differentiate into macrophages. These macrophages ingest lipids in an unrestricted manner via scavenger receptors, ultimately transforming into foam cells. These foam cells not only constitute the lipid core of neoatherosclerosis but also secrete abundant pro-inflammatory cytokines (e.g., IL-1, IL-6, TNF-α) and matrix metalloproteinases (MMPs). This microenvironment stimulates the aberrant proliferation and migration of vascular smooth muscle cells (VSMCs) into the intima, thereby accelerating neointimal hyperplasia and vascular remodeling (10).(3) Endothelial repair impairment and coagulation cascade activation: Beyond cholesterol transport, HDL-C exerts cytoprotective effects, including promoting endothelial nitric oxide (NO) release, as well as anti-apoptotic and antioxidant activities. An elevated CRI-II often reflects a relative or absolute deficiency in HDL-C functionality. Consequently, the endothelium damaged post-PCI is deprived of timely repair, leaving the bare metallic struts exposed chronically. This persistent exposure triggers a localized coagulation cascade and a deleterious inflammatory feedback loop, ultimately inducing ISR. Similarly, Endo et al. (11) evaluated the predictive value of CRI-II, albeit in a distinct cohort of patients with a history of PCI who developed CHD after a stable period of 6 to 12 months. Their study demonstrated that CRI-II serves as an independent predictor for both late target lesion revascularization and *de novo* lesion revascularization

Our study demonstrated that the composite lipid index (LCI) is significantly associated with coronary ISR. Furthermore, LCI exhibited superior predictive efficacy for this adverse event compared to single conventional lipid parameters, such as LDL-C or TG. This incremental predictive value stems from the dual superiority of LCI across both mathematical reconstruction and pathophysiological evaluation dimensions:(1)In clinical practice, a substantial proportion of patients undergoing PCI experience ISR despite intensive statin therapy that successfully lowers LDL-C to guideline-recommended targets (e.g., <1.8 mmol/L or even <1.4 mmol/L). Traditional predictive models often fall into the pitfall of over-relying solely on LDL-C. Conversely, LCI effectively identifies these high-risk patients who are otherwise masked by seemingly optimal LDL-C levels, thereby addressing the clinical blind spot of residual cardiovascular risk.(2) During post-PCI follow-up, many patients who develop ISR do not exhibit extreme lipid abnormalities; rather, their parameters hover at “borderline” or “mildly elevated” thresholds, which clinicians frequently overlook. LCI is highly sensitive to these concurrent, low-grade abnormalities. Mechanistically, the numerator of LCI employs a multiplicative formulation (TC*TG *LDL-C). Mathematically, multiplying multiple values that are each slightly above baseline results in a geometric compounding of the product. Concurrently, a depressed denominator (HDL-C), serving as a protective factor, further exacerbates this ratio (12). Consequently, LCI functions as an analytical amplifier, sensitively capturing and quantifying the cumulative lipotoxic potential generated by the synergy of multiple subtle pro-atherogenic fluctuations, thereby providing an early warning signal (13).(3) Beyond serving as a static predictive score, LCI inherently reflects the state of a dynamic lipid metabolic network. Compared to traditional atherogenic indices, such as the atherogenic index of plasma (e.g.,AIP), LCI offers a deeper and more comprehensive characterization of systemic lipid metabolic disequilibrium.

These findings offer pivotal clinical implications: for post-PCI patients, clinical management should transition away from an oversimplified focus on monolithic “LDL-C target attainment” toward the integration of LCI for comprehensive lipid profile surveillance. For patients presenting with an elevated LCI despite meeting guideline-recommended LDL-C targets, more aggressive, combination lipid-lowering strategies—such as the adjunctive administration of highly purified omega–3 fatty acids—warrant consideration (14). The PROMINENT trial, however, demonstrated that pemafibrate did not reduce cardiovascular events despite significant triglyceride lowering, indicating that the benefit of fibrates in this setting remains unestablished (15). Furthermore, holistic management targeting multiple metabolic nodes, including glucose metabolism and insulin resistance, is critically required to arrest the progression of *de novo* neoatherosclerosis within the stented segments.Future studies should explore whether incorporating CRI-II and LCI into comprehensive clinical scoring systems (combining mechanical and procedural factors) could yield higher prognostic accuracy.

The study has several noteworthy limitations. Firstly, it was retrospective and confined to a single center, potentially resulting in selection bias. No external validation or internal split-sample validation was performed to verify the robustness of the predictive model. We did not adjust for stent generation, stent diameter, stent length, or duration of dual antiplatelet therapy (DAPT) in the multivariate model. Secondly, in our multivariate logistic regression analysis, none of the novel systemic inflammatory indices (e.g., SII, SIRI, and PIV) retained an independent association with ISR, despite demonstrating significant differences in the univariate analysis.This finding should be interpreted with caution under several systemic limitations. First, all hematological parameters were quantified based on a single blood sample obtained within 24 h prior to PCI. Because ISR is a protracted, dynamic pathological process driven by chronic local vascular inflammation, neointimal hyperplasia, and vascular remodeling over months or years, a single pre-procedural snapshot is inherently limited and fails to capture the dynamic trajectories and fluctuations of these inflammatory markers during the post-PCI follow-up period.Furthermore, although we meticulously excluded individual blood cell counts from the multivariate models to mitigate structural multicollinearity, these combined inflammatory indices inherently mirror the patient's systemic immune-inflammatory burden and complex clinical overlapping states. Consequently, their genuine predictive signals might have been attenuated or masked by other potent, well-established clinical adjusting variables (such as age, creatinine, and fasting blood glucose) within the regression equations. Finally, due to the retrospective, single-center design of this study, residual confounding from unmeasured factors—such as variations in pre-admission medication adherence (e.g., antiplatelet or anti-inflammatory drugs) or subclinical infections—cannot be entirely ruled out.

## Conclusion

In conclusion, this study highlighted significant associations between ISR post-PCI and the non-traditional lipid parameters CRI-II and LCI. Among these markers, CRI-II showed high sensitivity, potentially functioning as an effective screening measure for identifying patients at heightened risk. Although their individual predictive capacities are moderate, these readily available indices offer valuable supplementary information for early ISR risk stratification, with important clinical implications. Future multicenter, prospective studies with regular postoperative non-traditional lipid parameters monitoring are warranted to determine whether dynamic trajectories have greater predictive value than single measurements.

## Data Availability

The raw data supporting the conclusions of this article will be made available by the authors, without undue reservation.
